# Food Disgust Scale: Spanish Version

**DOI:** 10.3389/fpsyg.2020.00165

**Published:** 2020-02-07

**Authors:** Leonor García-Gómez, César Romero-Rebollar, Christina Hartmann, Michael Siegrist, Guillaume Ferreira, Ruth Gutierrez-Aguilar, Salvador Villalpando, Gustavo Pacheco-Lopez

**Affiliations:** ^1^Health Sciences Department, Metropolitan Autonomous University (UAM), Campus Lerma, Lerma, Mexico; ^2^Department of Research in Smoking and COPD, National Institute of Respiratory Diseases (INER) Ismael Cosio Villegas, Mexico City, Mexico; ^3^Department of Health Sciences and Technology, Swiss Federal Institute of Technology (ETH) Zurich, Zurich, Switzerland; ^4^NutriNeuro, UMR INRA 1286, University of Bordeaux, Bordeaux, France; ^5^Research Division, School of Medicine, National Autonomous University of Mexico (UNAM), Mexico City, Mexico; ^6^Laboratory of Metabolic Diseases: Obesity and Diabetes, Children’s Hospital of Mexico (HIM) Federico Gómez, Mexico City, Mexico; ^7^Department of Gastroenterology & Nutrition, Children’s Hospital of Mexico (HIM) Federico Gómez, Mexico City, Mexico

**Keywords:** Food Disgust Scale, Mexico, disgust sensitivity, aversion, picky eating, BMI

## Abstract

**Introduction:**

The Food Disgust Scale (FDS) was recently developed and validated in Swiss adult population. This study aims to: (1) validate the FDS for the first time in a Spanish-speaking Mexican population, (2) correlate food disgust sensitivity with picky eating measures, and (3) explore the association between food disgust sensitivity and body mass index (BMI).

**Materials and Methods:**

A Spanish version of the FDS (FDS-Sp) and its short version (FDS-Sp short) were tested with confirmatory factor analysis (CFA) in order to test the original item/factor structure. Bivariate correlations were performed to determine the association between FDS-Sp/FDS-Sp short scores and picky eating. Lastly, hierarchical linear regression analysis was carried out to determine the relationship between food disgust sensitivity and BMI.

**Results:**

The factor structure of the FDS was replicated and acceptable internal consistency values were observed for FDS-Sp subscales (α varied between 0.781 and 0.955). Moreover, FDS-Sp subscales and FDS-Sp short were correlated with picky eating. Higher score in VEGI subscale of the FDS-Sp was a significant predictor for higher BMI, explaining 4% of the variance.

**Conclusion:**

FDS-Sp is a useful, reliable and robust psychometric instrument to measure the sensitivity to unpleasant food situations in a Mexican adult Spanish-speaking population. A relationship between food disgust sensitivity and picky eating, selective eating behaviors and neophobia in Mexicans was confirmed. BMI is multifactorial and only one subscale of FDS-Sp is a significant predictor for BMI status. These results are helpful to continue exploring food disgust in diverse populations.

## Introduction

Disgust is one of the basic human emotions that shoot up quickly as a reflex response to protect us from a potentially toxic/infectious exposure. Disgust mainly relays on sensory inputs from smell and/or taste, but also it is possible to trigger it by visual and vestibular stimulation (Celeghin et al., 2017). Particularly, food disgust is related to the avoidance of pathogens in food (Curtis et al., 2011). Then, it could lead to avoidance behaviors and also to selective eating patterns (Brown et al., 2013).

Predation and food intake tend to follow stable patterns, since exposure to new unfamiliar food sources represents a potential health threat. This leads individuals to reject unfamiliar and new foods to an innate food neophobia as an adaptive survival mechanism (Brown et al., 2013). Usually, food neophobia decreases from childhood to adolescence (Dovey et al., 2008).

On the other hand, non-spoiled food that is unfamiliar may provoke aversion and avoidance to a wide variety of commonly accepted foods; what is known as picky eating (Kauer et al., 2015). Often the rejection is related to organoleptic properties of food (Dovey et al., 2008; Kauer et al., 2015). Picky eating may be present either in childhood and adulthood, and literature provides diverse causes, among them, traits of avoidant/restrictive food intake disorder (ARFID) in Diagnostic and Statistical Manual of Mental Disorders (DSM-5) or Obsessive Compulsive Disorder (OCD) (Kauer et al., 2015; Thompson et al., 2015; Brown et al., 2016).

Both, picky eating and food neophobia are correlated with food disgust sensitivity (Lafraire et al., 2016). Consequently, food disgust sensitivity seems to play an important role in eating behavior (Brown et al., 2016; Van Tine et al., 2017). In this regard, restrictive eating behavior driven by strong food disgust sensitivity may interfere with taking risks in trying new or varied food.

Having a varied diet increases the likelihood of greater nutrient intake. In contrast, people with extreme picky eating behaviors who meet criteria for ARFID can even suffer from nutritional deficiencies (Fisher et al., 2014). It is still unknown if people with picky eating, but who do not meet the other criteria for this disorder (e.g., anorexia nervosa or bulimia nervosa), may also have nutritional deficiencies. Adult picky eaters often dislike fruits and vegetables, and prefer instead palatable and energy-dense foods (Thompson et al., 2015; Zickgraf and Schepps, 2016). Studies in children and adolescents have described that picky eating is associated with overweight (Brown et al., 2016; Ellis et al., 2018). However, for adults the relationship between picky eating and overweight/adiposity is not clear yet.

Interestingly, Watkins et al. (2016) found a lower proneness to disgust in obese people in comparison with normal weight people (Watkins et al., 2016). Obese participants also exhibited less activity of the amygdala in functional magnetic resonance imaging (fMRI) task when observing images of contaminated food. The authors suggest that low food disgust sensitivity may be related to larger amount of calories ingested.

Differences among individuals may be due by habituation to normalize some food cultural practices, cognition (risk perception vs. innocuity) and protective mechanisms acquired ontogenetically by individual experiences (e.g., gut microbiota enterotype, adaptive immunity) (Tybur et al., 2018). Also, subjects may categorize the ingestion of certain food as aversive by associative learning processes; developing conditioned taste avoidance/aversion to particular organoleptic food features associated to noxious postprandial experiences (Bermúdez-Rattoni, 2004).

Recently, the Food Disgust Scale (FDS) was developed to specifically measure sensitivity to food related situations that may threaten health (Hartmann and Siegrist, 2018). This psychometric instrument assesses an individual’s tendency to react with disgust in certain food-related situations that indicate for example unhygienic behavior at kitchen, food contamination, and food decaying. Food disgust sensitivity is related to other constructs of food behavior such as food neophobia. Measuring the sensitivity to food disgust might help to understand the factors that drive the acceptance and rejection of aversive situations related to food intake, and thus, consumers’ food choices. Food disgust has different dimensions that can vary between populations or demographic groups (Curtis et al., 2011), and so far, there is little information available on these differences. FDS was originally developed for, and validated in a European population, but it has not been explored in other cultures and environments. Therefore, the present study aims to: (1) validate the FDS for the first time in the Spanish-speaking Mexican population, (2) correlate food disgust sensitivity with picky eating measures, and (3) explore the association between food disgust sensitivity and body mass index (BMI).

## Materials and Methods

### Participants

Volunteers were Mexican adults recruited through social media (Facebook^®^, Twitter^®^ and WhatsApp^®^) during February 21 to March 4, 2018. Recruitment was voluntary, and no monetary incentives were given to the participants. Although no location was registered, it is assumed that most of the volunteers are urban residents of the metropolitan area of Mexico City.

### Instruments

FDS was developed and validated in Swiss adult population (Hartmann and Siegrist, 2018). It comprises 32 items, in which a Likert-type scale from 1 to 6 is scored on how disgusting a food-related situation is. The items are divided into eight subscales: (1) animal meat (MEAT) – situations associated with raw meat or certain parts of animals, (2) poor hygiene (HYG) – poor hygienic conditions in the preparation of food or eating, (3) human contamination (HUCON) – shared use of cutlery or other people’s contact with utensils and food, (4) mold (MOLD) – mold that has been removed from food, (5) decaying fruit (FRUIT) – fruits that are overripe and change their color or texture, (6) fish (FISH) – texture and smell of fish, (7) decaying vegetables (VEGI) – vegetables that are overripe and change their color or texture, and (8) living contaminants (LCON) – exposure of food to worms. In each factor the items are numbered consecutively (see Supplementary Tables 2, 3). The scale has been validated in a German-speaking Swiss adult population. Hartmann and Siegrist (2018) have also validated a short version of the FDS (Hartmann and Siegrist, 2018). The FDS short version takes 8 of the 32 items that had strong loads on the eight subscales. From the 32 items, it is possible to calculate the score of the short version. Back translation (Chen and Boore, 2010) of the original FSD was carried out to get the final version in Spanish (Supplementary Table 1). The items in the original short version of FDS were the same as those used for FDS-Sp-short.

Picky eating – Stanford Feeding Questionnaire (SFQ). Based on Toyama and Agras (2016) about persistent picky eating measuring, an adapted item of the SFQ was used: “Are you a picky eater?” (SFQ1) (Toyama and Agras, 2016). Additionally, selective eating behavior and food neophobia were assessed with the items “Do you have strong likes with regard to food?” (SFQ2), and “Do you accept new foods readily?” (SFQ3). Those three items were scored in a Likert-type scale from 1 (totally disagree) to 6 (totally agree) in order to make correlations analysis between SFQ and FDS-Sp score. Toyama and Agras (2016) described that the item “Are you a picky eater?” have proven to identify picky eaters throughout life and to discriminate persistent picky eaters (Toyama and Agras, 2016).

### Anthropometric and Demographic Measurements

Additional data were also self-reported such as sex, age, educational level, weight and height.

### Data Collection

The final FDS-Sp was used in an electronic format using the Google^®^ Docs platform (Google, 2015). The Google forms link was distributed via social media.

### Statistical Analysis

Reliability analysis (Cronbach’s alpha) was performed for each subscale. In the original study, an exploratory factor analysis yielded an eight-factor model, and each subscale corresponds to each of the eight factors. In this study, the same eight-factor model was assumed with the version of 32 items (Hartmann and Siegrist, 2018), and it was verified through a confirmatory factor analysis (CFA) using a maximum likelihood estimation method. The fit of the model was examined through the Chi-squared test, the Root Mean Square Error of Approximation (RMSEA < 0.05), the comparative fit index (CFI > 0.95) and the normed fit index (NFI > 0.90). Modification indices were verified to identify redundant elements. For FDS-Sp-short, a CFA was also carried out with a maximum likelihood estimation method, and the same fit criteria were applied. To compare Mexican and Swiss population (original data from the study published by Egolf et al., 2018) independent samples *t*-tests were done between mean scores of each subscale and mean score of FDS-short by sex. To explore the sex differences in FDS-Sp subscales and FDS-Sp-short in Mexican sample, independent samples *t*-test were also carried out. In order to explore the relation between age and food disgust sensitivity, a series of bivariate Pearson’s correlations were carried out.

Bivariate Pearson correlations were calculated between the mean score of each FDS-Sp subscale and the raw score of each of the SFQ items. Partial correlation analysis corrected by age and sex was calculated additionally. Statistically significance was set at *p* < 0.01.

A hierarchical linear regression analyses was performed in two steps. The first one included mean score of each of the eight factors (MEAT, HYG, HUCON, MOLD, FRUIT, FISH, VEGI, and LCON) and FDS-Sp short mean score as predictive variables for BMI. For the second step, sex and age were added since these variables have been reported to have a significant effect on food disgust. Both models were compared using Δ*R*^2^ to test for significant change in outcomes’ explained variance. A *p*-value < 0.01 was set for determination of statistical significance.

IBM SPSS AMOS 24 was used for CFA and the rest of the analysis were done with SPSS 20 (BDSC Cat# 7008, RRID:BDSC_7008) (IBM Corporation, 2011).

## Results

Recruitment yielded 1,328 volunteers. In order to have a comparable sample with the FDS original study by Hartmann and Siegrist (2018), 119 cases of participants less than 20 years old were excluded (age mean [SD], Min–max = 34.94 [12.32], 20–78; years of education = 15.79 [2.2], 6–18; women *n* [%] = 778 [67.7%]). Data exploration identified that some BMI values were extreme, which could indicate error in the size/weight report, or some pathological condition that would place them with extreme obesity or low weight. Based on BMI distribution, outliers were detected by the “identify unusual cases algorithm” implemented in SPSS. Sixty subjects were excluded: 36 “underweight” (BMI Min–max: 14.69–18.56), 24 “obesity II, III” (BMI Min–max = 36.98–59.44). The final sample consisted of 1,149 persons. For the internal consistency analysis and the confirmatory factor analysis, the sample was randomly divided into two parts in order to have a sample size similar to that used in the original validation of the scale.

### Internal Consistency and Confirmatory Factor Analysis

**FIGURE 1:**
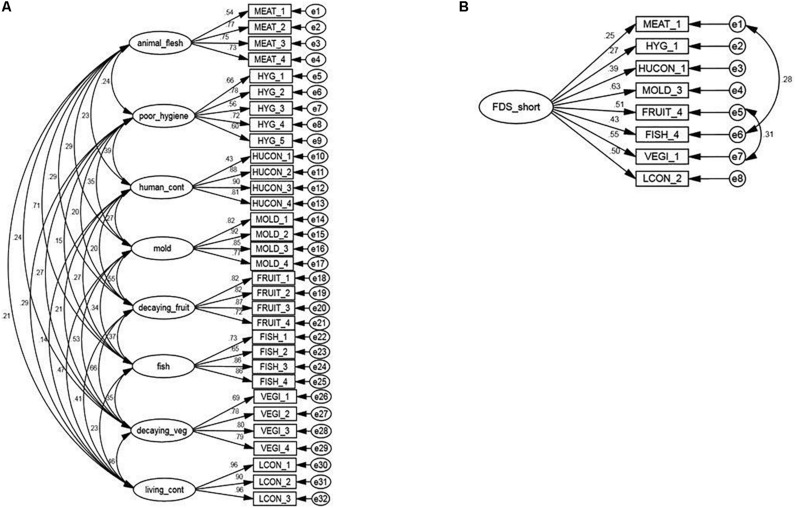
Confirmatory factor analysis models. **(A)** Eight-factor model for the Food Disgust Scale in Spanish (FDS-Sp). The CFA for 32-item version with eight subscales was consistent: CFI = 0.947, NFI = 0.912, RMSEA = 0.048, χ^2^ = 1020.434 (*p*-value < 0.001), df = 436. **(B)** Single factor model for the Food Disgust Scale in Spanish (FDS-Sp). CFA for the short version showed acceptable goodness-of-fit indices: CFI = 0.951, NFI = 0.925, RMSEA = 0.053, χ^2^ = 47.903 (*p*-value < 0.001), df = 18.

FDS-Sp reached acceptable internal consistency values for each of the eight subscales (MEAT α = 0.781, HYG α = 0.781, HUCON α = 0.840, MOLD α = 0.903, FRUIT α = 0.879, FISH α = 0.855, VEGI α = 0.850, LCON α = 0.955). The CFA for the eight-factor model had acceptable goodness-of-fit indexes in the standardized solution (CFI = 0.947, NFI = 0.912, RMSEA = 0.048, χ^2^ = 1020.434, *p* < 0.001, df = 436). Short version of FDS-Sp reached an internal consistency value of α = 0.677. The CFA for the short version yielded the following goodness-of-fit indices: CFI = 0.827, NFI = 0.803, RMSEA = 0.095, χ^2^ = 125.761, *p* < 0.001, df = 20. Given the value of modification index between the measurement errors of items MEAT1 and FISH4, and also between FRUIT4 and VEGI1, it is inferred that these pairs of items are highly correlated. The CFA for the short version adding co-variables showed better goodness-of-fit indices: CFI = 0.951, NFI = 0.925, RMSEA = 0.053, χ^2^ = 47.903, *p* < 0.001, df = 18. The factor models of the 32-item version and the one-factor model of the short version are shown in Figures 1A,B (*n* = 586; Supplementary Tables 2, 3).

Independent samples *t*-test showed significant (*p* < 0.01) differences between Swiss and Mexican populations in the subscales of HYG, FRUIT, FISH, VEGI, and LCON, with the Mexican population having the higher scores in all those subscales, except in FISH (Table 1). When the *t*-tests were made by sex, the same significant differences were maintained in FISH and VEGI subscales. When comparing the FRUIT subscale, Mexican women scored higher than Swiss women. And, on the other hand, Mexican men were more sensitive in LCON compared to Swiss men.

**TABLE 1:** Mean values, standard deviations, and *t*-test values for the eight subscales and the short version of FDS by sex.

	CH (*n* = 527) *M*(SD)	MEX (*n* = 1149) *M*(SD)	*t*-test	CH male (*n* = 258) *M*(SD)	MEX male (*n* = 371) *M*(SD)	*t*-test	CH female (*n* = 269) *M*(SD)	MEX female (*n* = 778) *M*(SD)	*t*-test
MEAT	2.75 (1.29)	2.92 (1.39)	−2.30	2.34 (1.15)	2.25 (1.17)	0.93	3.14 (1.30)	3.23 (1.38)	−0.92
HYG	5.21 (0.86)	5.36 (0.79)	−3.35*	4.98 (0.96)	5.15 (0.92)	−2.21	5.44 (0.69)	5.46 (0.71)	−0.43
HUCON	2.93 (1.19)	2.98 (1.34)	−0.65	2.89 (1.18)	2.82 (1.32)	0.64	2.97 (1.19)	3.05 (1.35)	−0.84
MOLD	4.23 (1.48)	4.25 (1.53)	−0.17	4.03 (1.52)	4.05 (1.52)	−0.18	4.43 (1.42)	4.34 (1.52)	0.82
FRUIT	2.64 (1.17)	3.14 (1.41)	−7.03*	2.62 (1.15)	2.77 (1.31)	−1.52	2.66 (1.19)	3.31 (1.42)	−6.69*
FISH	3.21 (1.51)	2.61 (1.45)	7.79*	2.92 (1.42)	2.16 (1.29)	6.92*	3.49 (1.54)	2.82 (1.48)	6.35*
VEGI	3.32 (1.26)	3.77 (1.37)	−6.48*	3.13 (1.24)	3.56 (1.36)	−3.98*	3.50 (1.26)	3.88 (1.36)	−4.04*
LCON	4.82 (1.29)	5.08 (1.35)	−3.67*	4.56 (1.37)	4.86 (1.50)	−2.49*	5.07 (1.16)	5.19 (1.25)	−1.32
FDS short	3.75 (0.93)	3.86 (0.90)	−2.28	3.52 (0.94)	3.56 (0.89)	−0.52	3.97 (0.86)	4.00 (0.87)	−0.53

CH, Swiss; MEX, Mexico. FDS subscales: MEAT = animal flesh, HYG = poor hygiene, HUCON = human contamination, MOLD = mold, FRUIT = decaying fruit, FISH = fish, VEGI = decaying vegetables, LCON = living contaminants, FDS short = FDS short (8 items). Possible scoring range 1 (not disgusting at all) to 6 (very disgusting). * *p* < 0.01.

For all FDS-Sp subscales, women obtained significant higher scores (*p* < 0.01). Also, for FDS-Sp short, difference was statistically significant (male: mean = 3.56 [0.89], female: mean = 4.00 [0.87], *t* [711.45] = −7.87, *p* < 0.01). Additionally, we found a weak positive correlations with age (HYG, *r* = 0.11; HUCON, *r* = 0.21; VEGI, *r* = 0.08, all *p* < 0.01) and negative correlations with age were found (MEAT, *r* = −0.12; MOLD *r* = −0.13; LCON, *r* = −0.13, *p* < 0.01) (Table 1).

### Correlation of FDS-Sp Scores With Picky Eating Measures

Correlations between the mean score of each FDS-Sp subscale and SFQ1 (“Are you a picky eater?”) were positive and statistically significant, as well, almost all for FDS-Sp mean score and SFQ2 (“Do you have strong likes with regard to food?”) (Table 2). Correlation between FDS-Sp mean score and SFQ3 (“Do you accept new foods readily?”) was negative and statistically significant, except for one subscale. Correlations between FDS-Sp short mean score and 3 items of SFQ were all significant. Given that differences between sex and significant correlations with age were found, we made partial correlation analysis corrected by age and sex; all the correlations remained significant, except HYG and HUCON correlations with SFQ1 and SFQ2.

**TABLE 2:** Bivariate correlations and partial correlations between picky eating items (SFQ) and FDS-Sp subscales scores controlling by sex and age (*n* = 1149).

Partial correlation analysis corrected by age and sex													
Control variables		SFQ1	SFQ2	SFQ3	MEAT	HYG	HUCON	MOLD	FRUIT	FISH	VEGI	LCON	FDS-Sp short
None^a^	SFQ1												
	SFQ 2	0.467*											
	SFQ 3	−0.295*	−0.145*										
	MEAT	0.360*	0.237*	−0.293*									
	HYG	0.143*	0.090*	−0.083*	0.215*								
	HUCON	0.191*	0.065	−0.092	0.219*	0.394*							
	MOLD	0.285*	0.170*	−0.205*	0.292*	0.333*	0.312*						
	FRUIT	0.235*	0.152*	−0.184*	0.249*	0.215*	0.282*	0.480*					
	FISH	0.357*	0.225*	−0.335*	0.576*	0.163*	0.304*	0.320*	0.340*				
	VEGI	0.194*	0.122*	−0.138*	0.226*	0.294*	0.291*	0.467*	0.635*	0.328*			
	LCON	0.181*	0.181*	−0.142*	0.230*	0.297*	0.160*	0.452*	0.378*	0.233*	0.432*		
	FDS-Sp	0.408*	0.272*	−0.319*	0.583*	0.455*	0.467*	0.678*	0.668*	0.639*	0.677*	0.574*	
Age and sex^b^	SFQ 1												
	SFQ 2	0.463*											
	SFQ 3	−0.293*	−0.142*										
	MEAT	0.350*	0.226*	−0.284*									
	HYG	0.146*	0.088	−0.069	0.184*								
	HUCON	0.213*	0.076	−0.086	0.237*	0.371*							
	MOLD	0.272*	0.160*	−0.202*	0.269*	0.346*	0.349*						
	FRUIT	0.227*	0.144*	−0.172*	0.206*	0.190*	0.280*	0.478*					
	FISH	0.350*	0.217*	−0.326*	0.549*	0.135*	0.309*	0.310*	0.315*				
	VEGI	0.197*	0.122*	−0.130*	0.215*	0.272*	0.273*	0.480*	0.631*	0.318*			
	LCON	0.165*	0.170*	−0.136*	0.193*	0.305*	0.188*	0.437*	0.639*	0.214*	0.442*		
	FDS-Sp	0.400*	0.264*	−0.310*	0.550*	0.442*	0.487*	0.679*	0.656*	0.621*	0.648*	0.566*	

^*a*^*N* = 1149, ^*b*^df = 1145, * *p* < 0.0. SFQ1 = “Are you a picky eater?”, SFQ 2 = “Are you a picky eater?”, SFQ 3 = “Do you accept new foods readily?”; FDS subscales: MEAT = Animal flesh, HYG = poor hygiene, HUCON = human contamination, MOLD = mold, FRUIT = decaying fruit, FISH = fish, VEGI = decaying vegetables, LCON = Living contaminants, FDS short = FDS short (eight items).

### Predictive Validity of FDS-Sp for BMI

Hierarchical linear regression was calculated to predict BMI based on each factor of FDS-Sp in the first model. Age and sex were added in the second model (Table 3). In the first model, a significant regression equation was found (*F* [8, 1140] = 5.39, *p* < 0.01), with a R^2^ of 0.036. In the second model, also a significant regression equation was found (*F* [2,1138] = 11.20, *p* < 0.01), with R^2^ of 0.090, being significantly higher than the first model (Δ*R*^2^ = 0.053, *p* < 0.01). Also, in the first model, the variables MEAT, FRUIT, and VEGI predicted 4% of the variance in BMI. Higher scores on the subscales of MEAT and FRUIT predicted lower BMI, while higher scores on the subscale of VEGI predicted higher BMI. Adding age and sex in the second model, the variance explained increased to 9%. However, except for the VEGI subscale, none of the other subscales of the FDS-Sp were significant predictors of BMI anymore when simultaneously including sex and age in the model. Thus, there is a tendency for the more disgust sensitivity for vegetable related cues, the higher the BMI might be.

**TABLE 3:** Hierarchical linear regression analyses testing effect of FDS-Sp subscales, sex and age on BMI (*n* = 1149).

Models	Measures	Unstandardized coefficient		*p*	*F*	*R*^2^	Δ*R*^2^
		*B*	SE				
1					5.39	0.04	
	Constant	26.64	0.77	<0.01			
	**MEAT**	−0.37	0.09	<0.01			
	HYG	−0.24	0.15	0.12			
	HUCON	0.14	0.09	0.11			
	MOLD	0.08	0.09	0.36			
	**FRUIT**	−0.32	0.10	<0.01			
	FISH	0.21	0.09	0.02			
	**VEGI**	0.45	0.11	<0.01			
	LCON	−0.17	0.09	0.07			
2					11.20	0.090	0.053
	Constant	26.77	0.84	<0.01			
	MEAT	−0.17	0.09	0.08			
	HYG	−0.18	0.15	0.22			
	HUCON	0.01	0.09	0.85			
	MOLD	0.11	0.09	0.19			
	FRUIT	−0.23	0.10	0.02			
	FISH	0.22	0.09	0.016			
	**VEGI**	0.34	0.10	<0.01			
	LCON	−0.10	0.09	0.27			
	**Sex**	−1.57	0.24	<0.01			
	**Age**	0.04	0.009	<0.01			

*MEAT = animal meat; HYG = poor hygiene; HUCON = human contamination; MOLD = mold; FRUIT = decaying fruit; FISH = fish; VEGI = decaying vegetables; LCON = living contamination.*

## Discussion

The FDS-Sp eight-factor model replicates in adult Mexican population with similar values as in the original FDS version developed with Swiss adults: both internal consistency values and goodness-of-fit indexes for the 8-factor model and short version are acceptable and similar to those originally reported (Hartmann and Siegrist, 2018). The parameters to accept the validity of the FDS-Sp are the values obtained from CFI and RMSEA. We would have expected that the Chi-squared would not be significant in the analysis. The reason for the misfit could be the large sample size, which, while reducing it, was still large for this type of analysis. However, our values of goodness of fit allow us to suggest that FDS-Sp is a useful psychometric instrument to measure the sensitivity to unpleasant situations related to food in the Spanish-speaking Mexican adult population. The short version of FDS-Sp did not reached good internal consistency and good values of goodness of fit were reached adding co-variables in CFA. These correlations may have theoretical sense since the first pair relates the sensation of textures in the mouth (MEAT1 and FISH4) and the second pair present situations about eating plant-based foods that have oxidized and darkened (FRUIT4 and VEGI1); therefore, it can be said that each pair share a source of common variance. The replicability of the short version of the scale is not satisfactory. Therefore, it may be more useful to use the 32-item version to assess food disgust sensitivity in its eight factors.

Small, but statistically significant differences were found in the mean values of some FDS subscales between populations, with Mexicans reporting higher scores in VEGI, FRUIT (only female) and LCON (only male). Given the Mexican sample has more proportion of women, we analyze separately the subsamples of men and women, and differences remained. One might speculate that the lower FDS scores of the Swiss population may be related to social and cultural aspects. For instance, it is possible that there is greater societal confidence toward food quality control and sanitary regulation in Switzerland in comparison to Mexico. The Mexican population may feel that some foods are not safe because, compared to Switzerland, there is a higher rate of infectious gastrointestinal diseases and foodborne illnesses. In this regard, epidemiological data shows higher vulnerability to gastrointestinal infections in Latin American than in European countries (Fletcher et al., 2013). Besides, there are reports that reveal that in Mexico there are economic barriers to the implementation of food safety standards compared to European countries (Weinroth et al., 2018). Prokop et al. (2010) have found differences in disgust sensitivity when comparing countries that have different pathogen risk (Prokop et al., 2010). They measured self-perceived health and perceived danger, fear, and disgust of potentially hazardous parasites in children of Turkey and Slovakia. They found that better self-perceived health of children was associated with lower perceived pathogen risk, and that in Turkey (where there is higher prevalence of parasites) children exhibited greater emotions of fear and disgust, as well as reported greater precautionary behaviors against infections (Prokop et al., 2010). These findings are similar to ours, in terms that Mexico is a country with greater risk of foodborne illnesses and the population exhibit greater food disgust sensitivity than in Switzerland.

Interestingly, the only subscale in which Mexicans on average obtained lower FDS scores than Swiss and which Mexicans considered least disgusting was the FISH subscale. This might indicate that Mexican adults seem more tolerant to disgusting situations related to fish consumption. According to statistics of Food and Agriculture Organization (FAO), the annual per capita consumption of fish meal in 2013 in Switzerland was 17.78 kg, whereas in Mexico was 10.46 kg (FAO, 2013). Regardless of the difference between consumption in both countries, it is possible that the Mexican population is more accustomed to fish-related “offensive” situations since Mexico has extensive seashore on both Pacific and Atlantic oceans, while Switzerland is a landlocked country. Geographical conditions may be related to greater exposure and habituation to what involves unprocessed fish. In other study conducted by Kunst and Palacios-Haugestad (2018) they compared willingness to eat meat in American and Ecuadorian adults (Kunst and Palacios-Haugestad, 2018). They found that, the level of disgust and empathy for the killed animal are predictors of less willingness to eat meat. However, continuous exposure to cues that link meat to animal origins (i.e., the head of pork roast) increases the ability to dissociate these animal origins from meat meal and increase willingness to eat meat. This was tested with hypothetical situations about eating unprocessed meat food. This may happen because in their country it is more common to observe these cues that link to animal origins, and therefore, they can dissociate better animal origins of meat food (Kunst and Palacios-Haugestad, 2018). Even though in our study we are not measuring willingness to eat fish, Kunst and Palacios-Haugestad findings could be useful to explain why Mexican population obtained less score in FISH subscale, assuming that Mexican population is more exposed to unprocessed fish due to its geographical conditions and culinary traditions. It would be interesting to systematically explore if there is a relationship between cultural determined consumption habits and the manifestation of certain kinds of disgust sensitivity.

In the Mexican sample, we found differences between sexes for each subscale and in the short version, with women obtaining higher scores. In the study of Egolf et al. (2018), women also showed higher FDS scores than men (Egolf et al., 2018). The observed sexual difference could be related to the evolutionary role of females in reproduction, since there is a prolonged physiological interaction between mother and baby and greater protection is required (Al-Shawaf et al., 2018). Therefore, the prevention of infections via contact with potential foodborne pathogens is of high importance for women.

Egolf et al. (2018) reported significant correlations between age and FDS scores: age was positively correlated with FDS scores, indicating that older people had higher FDS scores than younger people (Egolf et al., 2018). In the present data, HYG, HUCON, and VEGI subscales also were positively associated with age. However, we also found negative correlations for MEAT, MOLD, LCON, and FDS-Sp short; the opposite of what they found (Egolf et al., 2018). Supporting our findings, Berger and Anaki (2014) reported that the effect of age varied according to the investigated disgust domain. Young people might react with greater aversion to the food situations described in the subscales of MEAT, MOLD, LCON and have greater scores in FDS-SP short due to an innate response that gets lost with repeated exposure during, for instance, aging. We hypothesize that gradual exposure (i.e., habituation) in safe conditions to “contaminated” food situations might desensitized people (e.g., eating sushi, gourmet mature cheese or steak tartare), and therefore with age there is a reduced disgust response. Humans have developed a preference for flavors and foods that we would not naturally ingest, but that confer variety on the intestinal microbiota, on the digestive mechanisms and improve defense against pathogens (Krebs, 2009). However, we recognize that our correlations are small, so they can only be taken as a hint.

The SFQ was used as an external criterion of disgust construct validity. It has been suggested that picky eating is an encompassing construct, and that only some picky eaters have high sensitivity to the disgusting situations that arise in the FDS. Food disgust and picky eating can be taken as constructs that seem to share similar underlying psychological drivers, and therefore, they may have convergent validity. The positive correlation between all FDS-Sp subscales and SFQ1 yielded the expected result: the greater the pickiness, the larger sensitivity to disgusting situations related to food. The SFQ2 item measures how selective a person is when eating, then, it is likely that people who score high on SFQ2 have little variety in the foods in their diet. High disgust sensitivity is associated with more restrictive eating behavior, and in fact, picky eaters often describe themselves as unhealthy eaters (Kauer et al., 2015; Egolf et al., 2018). Poor hygiene and human contamination were not significantly associated. Our argument is that these FDS subscales do not measure situations related to the intrinsic characteristics of food (texture, smell, taste or consistency) but of conditions related to food handling. And also, as expected, SFQ3 correlated negatively with many FDS-Sp subscales, except with HUCON and HYG. Maybe we must consider the neophobia construct separately. Acceptance of a new food is independent of aseptic conditions. While FDS does not explicitly assess willingness to try new foods, the negative correlation suggests that most sensitive tend to be more neophobic. Although the correlations between the SFQ items and the FDS-Sp subscales scores were not very high, many of them were statistically significant (Table 2). Egolf et al. (2018) also reported a relationship between picky eating and willingness to accept foods with varied textures (Egolf et al., 2018).

Conclusively, initially we hypothesized that there might be a positive correlation between BMI and food disgust. This question arose from studies that show association between picky eating and BMI in adolescents (Brown et al., 2016; Ellis et al., 2018) and the study of Watkins et al. (2016) that describe a lower proneness to disgust in obese people. Our study yielded that only the VEGI subscale contributes sparingly to the variance in BMI. It has been described that fruit and vegetable consumption throughout life is associated with low BMI and weight loss (Charlton et al., 2014; Yuan et al., 2018; Gaylis et al., 2019; Jones et al., 2019). Also, Sadeghi et al. (2019) found that an intervention with increased vegetable intake was associated with decrease in BMI in Mexican-heritage children (Sadeghi et al., 2019). High prevalence of childhood obesity might be related to very low vegetable consumption (20% in any given day) in Mexican children as shown by Deming et al. (2015). In our study, the contribution of the VEGI subscale to the variance in BMI is very small (4%). However, it is important to acknowledge that BMI is multifactorial (Löffler et al., 2017; McKey et al., 2017), thus is difficult for a single factor to explain a large variance percentage. Although it might be possible that food disgust can contribute to adiposity in some populations, only the VEGI subscale was able to predict the increase in BMI in urban Mexican adults.

Finally, we identified the following limitations in our study: it would be relevant to have the validity of the total scale score in order to make correlations with the other constructs. For now, we only perform the analyzes with the scores of each of the eight subscales, which correspond to the eight factors. Another limitations are that: (a) the data was collected via Internet; (b) the sample characteristics were self-reported, so we cannot confirm age, sex, weight and height; (c) 67.7% of our sample were females, which can be a bias, since it has been shown the sensitivity of disgust to food differs between sexes. Also, we assume that most of the participants are from a Mexico City metropolitan area, but we don’t design any item to assess urban vs. rural category. Finally, it would have been interesting to know the socioeconomic income variable, as it is likely that this variable also has an influence on the sensitivity to disgust, as has been reported before (Egolf et al., 2018).

## Conclusion

Our findings document FDS as a useful psychometric instrument to measure the sensitivity to unpleasant situations related to food in a Mexican adult population. The food disgust construct, measured by FDS, mainly stands beside the language, cultural, and culinary differences. Thus, we argue that the FDS can be also extrapolated to other societies; however, further replications in other geographical and cultural contexts would increase its validity and reliability. Furthermore, as the comparison between Mexico and Switzerland showed differences in food disgust sensitivity, we encourage further exploration of the construct in other countries-cultures. We confirmed that there are food disgust differences between sexes, and it correlates with age. Also, we conclude that food disgust and picky eating are convergent concepts. Finally, the VEGI subscale was modestly predictive of BMI. Our results are helpful to continue exploring food disgust in diverse cultures.

## Data Availability Statement

The datasets for this study can be found in the XOGI Institutional Repository of the Metropolitan Autonomous University (UAM), Campus Lerma (http://hdl.handle.net/20.500.12222/244).

## Ethics Statement

This study was carried out in accordance with the principles and recommendations of the Official Mexican Standard NOM-012-SSA3-2012 related to research projects for human health. The study was reviewed and approved by the National Institute of Respiratory Disorders Ismael Cosio Villegas and the Children’s Hospital of Mexico (HIM) (approval code INER C41-17 and HIM 2017-59). The patients/participants provided their written informed consent to participate in this study.

## Author Contributions

LG-G coordinated the data collection along with CR-R, wrote the manuscript, and conducted the statistical analyses. CH and MS developed the original FDS and performed a critical review of the final version of the manuscript. GF, RG-A and SV advised the methodology and performed the critical review of the final version of the manuscript. GP-L conceptualized and coordinated the study as senior author, performed critical review of all versions of the manuscript, and managed international cooperation. All authors contributed to the final version of the manuscript and have approved the current version of the manuscript.

## Conflict of Interest

The authors declare that the research was conducted in the absence of any commercial or financial relationships that could be construed as a potential conflict of interest.
