# Supplementary material for: Food Disgust Scale: Spanish Version
Source: Front Psychol. 2020 Feb 7;11:165. doi: 10.3389/fpsyg.2020.00165 (PMC7020908; doi:10.3389/fpsyg.2020.00165)
Supplement: Supplementary file 1 [file Table_1.DOCX]

Supplementary Material

Supplementary Table 1. Food Disgust Scale - Spanish version (FDS-Sp) [ESCALA DE ASCO A LA COMIDA]

| **Instrucciones:**  Por favor marca qué tanto asco te producen las siguientes situaciones:  1 = Nada asqueroso  5 = Extremadamente asqueroso | | | | | |
| --- | --- | --- | --- | --- | --- |
| **Carne animal** | | | | | |
| 1. Introducir cartílago animal en mi boca | 1 | 2 | 3 | 4 | 5 |
| 2. Ver carne cruda | 1 | 2 | 3 | 4 | 5 |
| 3. Comer carne que aún tiene sangre por dentro | 1 | 2 | 3 | 4 | 5 |
| 4. Ver un cerdo entero rostizándose en brocheta | 1 | 2 | 3 | 4 | 5 |
| **Mala higiene** | | | | | |
| 5. Comer con cubiertos sucios en un restaurante | 1 | 2 | 3 | 4 | 5 |
| 6. Una comida preparada por un cocinero que tiene el cabello grasoso y las uñas sucias | 1 | 2 | 3 | 4 | 5 |
| 7. Si el cocinero de un restaurante tiene una herida abierta | 1 | 2 | 3 | 4 | 5 |
| 8. Si la gente se suena la nariz antes de servir mi comida | 1 | 2 | 3 | 4 | 5 |
| 9. Un pelo de otra persona en mi sopa | 1 | 2 | 3 | 4 | 5 |
| **Contaminación humana** | | | | | |
| 10. Comida regalada por un vecino que apenas conozco | 1 | 2 | 3 | 4 | 5 |
| 11. Si un amigo muerde mi pan | 1 | 2 | 3 | 4 | 5 |
| 12. Beber del mismo vaso en el que ha bebido un amigo | 1 | 2 | 3 | 4 | 5 |
| 13. Si amigos o conocidos han tocado mi comida | 1 | 2 | 3 | 4 | 5 |
| **Moho** | | | | | |
| 14. Comer la parte sin moho de un tomate mohoso | 1 | 2 | 3 | 4 | 5 |
| 15. Comer parte de un pan al que se le cortó un pedazo con moho | 1 | 2 | 3 | 4 | 5 |
| 16. Comer de un queso al cuál se le cortó la parte con moho | 1 | 2 | 3 | 4 | 5 |
| 17. Comer de una mermelada a la que se le ha removido el moho de la superficie | 1 | 2 | 3 | 4 | 5 |
| **Fruta en descomposición** | | | | | |
| 18. Comer frutas demasiado maduras | 1 | 2 | 3 | 4 | 5 |
| 19. Comer un plátano que tiene manchas negras | 1 | 2 | 3 | 4 | 5 |
| 20. Comer frutas mallugadas (ej. duraznos o manzanas) | 1 | 2 | 3 | 4 | 5 |
| 21. Comer rebanadas de manzana que se pusieron de color café por la exposición al aire | 1 | 2 | 3 | 4 | 5 |
| **Pescado** | | | | | |
| 22. Tener un pescado entero con cabeza en mi plato | 1 | 2 | 3 | 4 | 5 |
| 23. Comer pescado crudo como en el sushi | 1 | 2 | 3 | 4 | 5 |
| 24. Oler el pescado fresco en una pescadería | 1 | 2 | 3 | 4 | 5 |
| 25. La textura de algunos tipos de pescados en mi boca | 1 | 2 | 3 | 4 | 5 |
| **Vegetales en descomposición** | | | | | |
| 26. Comer un aguacate que se ha puesto café | 1 | 2 | 3 | 4 | 5 |
| 27. Comer un pepino tan maduro que se dobla porque está aguado | 1 | 2 | 3 | 4 | 5 |
| 28. Comer rábanos maduros que ya se encogieron | 1 | 2 | 3 | 4 | 5 |
| 29. Comer lechuga o ensalada que ya no está crujiente | 1 | 2 | 3 | 4 | 5 |
| **Contaminantes vivos** | | | | | |
| 30. Hay un gusano en la fruta que quería comer | 1 | 2 | 3 | 4 | 5 |
| 31. Hay un gusano en la ensalada que quería comer | 1 | 2 | 3 | 4 | 5 |
| 32. Hay un gusano en mi manzana | 1 | 2 | 3 | 4 | 5 |

| Supplementary Table 2. Standardized loadings of 32-item Food Disgust Scale Spanish version (n=586) | | | |
| --- | --- | --- | --- |
|  | Label | Item | Standardized loading (CFA) |
|  | **Animal flesh** |  |  |
| 1 | MEAT1 | To put animal cartilage into my mouth | .537 |
| 2 | MEAT2 | To see raw meat | .772 |
| 3 | MEAT3 | To eat a steak that is still bloody inside | .745 |
| 4 | MEAT4 | To see a whole pig en brochette | .727 |
|  | **Poor hygiene** |  |  |
| 5 | HYG1 | To eat with dirty silverware in a restaurant | .657 |
| 6 | HYG2 | A meal prepared by a cook who has greasy hair and dirty fingernails | .777 |
| 7 | HYG3 | If the cook in a restaurant has an open cut | .565 |
| 8 | HYG4 | If people blow their nose before they serve my meal | .717 |
| 9 | HYG5 | Another person’s hair in my soup | .604 |
|  | **Human contamination** |  |  |
| 10 | HUCON1 | Food donated from a neighbor whom I barely know | .435 |
| 11 | HUCON2 | If a friend bites into my bread | .881 |
| 12 | HUCON3 | To drink from the same drinking glass a friend has already drunk from | .905 |
| 13 | HUCON4 | If friends or acquaintance have touched my food | .811 |
|  | **Mold** |  |  |
| 14 | MOLD1 | To eat the mold-free part of a moldy tomato | .822 |
| 15 | MOLD2 | To eat bread from which mold was cut away | .916 |
| 16 | MOLD3 | To eat hard cheese from which mold was cut off | .854 |
| 17 | MOLD4 | To eat marmalade from which mold was removed from the surface | .766 |
|  | **Decaying fruit** |  |  |
| 18 | FRUIT1 | To eat overripe fruits | .815 |
| 19 | FRUIT2 | To eat a banana that has black spots | .818 |
| 20 | FRUIT3 | To eat fruits (e.g., apple and peach) with pressure marks | .873 |
| 21 | FRUIT4 | To eat apple slices that turned brown when exposed to air | .720 |
|  | **Fish** |  |  |
| 22 | FISH1 | To have a whole fish with its head on the plate | .734 |
| 23 | FISH2 | To eat raw fish like sushi | .653 |
| 24 | FISH3 | The smell in a fish shop or in fish sections with fresh fish | .855 |
| 25 | FISH4 | The texture of some kinds of fish in the mouth | .862 |
|  | **Decaying vegetables** |  |  |
| 26 | VEGI1 | To eat brown-colored avocado pulp | .642 |
| 27 | VEGI2 | To eat an overripe cucumber that can already be bent | .785 |
| 28 | VEGI3 | To eat shrunken radishes | .804 |
| 29 | VEGI4 | To eat salad that is not crispy anymore | .792 |
|  | **Living contaminants** |  |  |
| 30 | LCON1 | There is a maggot in the cherry that I wanted to eat | .956 |
| 31 | LCON2 | There is a little snail in the salad that I wanted to eat | .900 |
| 32 | LCON3 | There is a worm in my apple | .955 |
| Note: CFA = Confirmatory factor analysis | | | |

| Supplementary Table 3. Standardized loadings of 8-item Food Disgust Scale Spanish version (n=586) | | | |
| --- | --- | --- | --- |
|  | Label | Item | Standardized loading (CFA) |
|  | **Animal flesh** |  |  |
| 1 | MEAT1 | To put animal cartilage into my mouth | .250 |
|  | **Poor hygiene** |  |  |
| 5 | HYG1 | To eat with dirty silverware in a restaurant | .270 |
|  | **Human contamination** |  |  |
| 10 | HUCON1 | Food donated from a neighbor whom I barely know | .391 |
| 16 | MOLD3 | To eat hard cheese from which mold was cut off | .630 |
|  | **Decaying fruit** |  |  |
| 21 | FRUIT4 | To eat apple slices that turned brown when exposed to air | .509 |
|  | **Fish** |  |  |
| 25 | FISH4 | The texture of some kinds of fish in the mouth | .426 |
|  | **Decaying vegetables** |  |  |
| 26 | VEGI1 | To eat brown-colored avocado pulp | .547 |
|  | **Living contaminants** |  |  |
| 31 | LCON2 | There is a little snail in the salad that I wanted to eat | .501 |
| Note: CFA = Confirmatory factor analysis | | | |
